# An integrated immunoinformatic approach to design a novel multiepitope chimeric vaccine against *Mycoplasma phocimorsus* as a causal agent of bloodstream infections

**DOI:** 10.3389/fimmu.2025.1719398

**Published:** 2025-12-05

**Authors:** Rongrong Yu, Ahmad Hasan, Muhammad Ibrahim, Wadi B. Alonazi, Li Bin

**Affiliations:** 1 State Key Laboratory of Rice Biology and Breeding, Ministry of Agriculture and Rural Affairs Key Laboratory of Molecular Biology of Crop Pathogens and Insect Pests, Zhejiang Key Laboratory of Biology and Ecological Regulation of Crop Pathogens and Insects, Institute of Biotechnology, Zhejiang University, Hangzhou, China; 2 College of Education, Zhejiang University of Technology, Hangzhou, China; 3 Department of Biosciences, COMSATS University Islamabad, Sahiwal, Pakistan; 4 Health Administration Department, College of Business Administration, King Saud University, Riyadh, Saudi Arabia

**Keywords:** *Mycoplasma phocimorsus*, reverse vaccinology, subtractive genomics, B-cell epitopes, drug targets, MD simulation

## Abstract

**Introduction:**

*Mycoplasma phocimorsus* is increasingly recognized as an emerging human pathogen, despite its primary association with marine mammals. It has recently been identified as a causative agent of bloodstream infections and sepsis, a major cause of mortality among hospitalized patients. To date, no approved vaccine is available against *M. phocimorsus*, underscoring the urgent need for preventive strategies.

**Methods:**

The current study was aimed at employing immunoinformatic approaches to design a vaccine based on multiple epitopes derived from the six core proteomic datasets of representative *M. phocimorsus* strains.

**Results:**

By subtractive genomics, we retrieved 3,576 nonredundant proteins from *M. phocimorsus* proteomes following only one putative immunoglobulin-blocking virulence outer membrane protein conserved in six strains. The epitopes derived from the putative immunoglobulin-blocking virulence protein exhibited promising features such as strong binding affinity, lack of allergenicity, nontoxic properties, high antigenicity scores, and excellent solubility. Moreover, these epitopes include nine linear B cell epitopes, eight MHC class I epitopes, and five MHC class II epitopes. In addition, adjuvants and linker molecules were successfully merged into a chimeric vaccine with significant immunogenicity and stimulation of both adaptive and innate immune responses. The promising potential of the selected vaccine candidates was further validated through their favorable physico-chemical characteristics, strong interaction with TLR-4, and stable performance in molecular dynamics simulations.

**Discussion:**

These results suggest that the putative immunoglobulin-blocking outer membrane virulence protein could effectively participate in activating the primary innate immune response, thereby serving as a strong foundation for subsequent adaptive immune activation. The proposed vaccine provides substantial basis for developing effective preventive and therapeutic measures against the zoonotic *M. phocimorsus*, whose association with sepsis, soft tissue, and respiratory infections, particularly in immunocompromised individuals emphasizes the crucial need for vaccine development.

## 1 Introduction

Bloodstream infection (BSI) is a significant global public health challenge with high mortality rates. Prompt and effective treatment is crucial, as delays in therapy can severely impact patient outcomes (1). BSI can complicate the progression of many severe community acquired infectious diseases. *Streptococcus pneumoniae*, *Staphylococcus aureus*, *Klebsiella pneumoniae*, and *Escherichia coli* are responsible for more than 70% of all BSIs (2, 3). *Pseudomonas aeruginosa* accounts for up to 5% of community-onset bloodstream infections, predominantly affecting patients with severe underlying health conditions and recent exposure to healthcare settings (4). Similarly, the incidence of BSIs caused by community-associated methicillin-resistant *S. aureus* (MRSA) has shown signs of stabilizing in recent years, not only in the United States but also in the majority of other endemic regions, after a marked increase during the early 2000s (5, 6). Additionally, the number of BSIs caused by Enterobacterales that produce extended-spectrum beta-lactamase (ESBL) is slowly increasing because these pathogens are very common in the community. The incidence of ESBL-producing isolates in *K. pneumoniae* and *E. coli* bloodstream infections resulting from urinary tract infections currently surpasses 5%, with some locations reporting levels as high as 20%, comparable to total bloodstream infection rates (7, 8).

In the context of *M. phocimorsus*, bloodstream infections are a potential concern, particularly because of its emerging zoonotic role. Although humans and animals coexist in a symbiotic relationship, animals can also serve as reservoirs of zoonotic pathogens, posing significant health risks to humans through the transmission of infectious diseases (9). *M. phocimorsus* is increasingly recognized as an emerging human pathogen, despite its primary association with marine mammals. *M. phocimorsus* can infect humans, especially those who have close contact with marine mammals, such as veterinarians, marine biologists, zookeepers, and workers in rehabilitation centers (10). Marine mammals, such as harbor seals, often carry *Mycoplasma* species. During the late 1970 s and early 1980 s, *M. Phocidae*, *M. phocacerebrale*, and *M. phocarhinis* were first isolated from harbor seals in New England, USA, following an epizootic pneumonia outbreak (11–13). Recently, scientists identified a new species of *Mycoplasma* called *M. phocimorsus*, which was isolated from Scandinavian patients who had septic arthritis or seal arthritis after coming into contact with seals (14). Treatment is challenging due to antibiotic resistance (13, 14). Moreover, a recent case reported a woman who developed tendinous panaritium following a cat scratch, from which *M. phocimorsus* was identified, suggesting the possibility of transmission through non-traditional animal hosts (15).

Vaccination is crucial for public health, controlling germ outbreaks and reducing mortality rates. Current vaccines are not universally used and require ongoing development and an understanding of immune response mechanisms (16–18). Pangenome sequencing analyses offer comprehensive insights into the antigenic repertoire of the target pathogen, while high-throughput approaches facilitate the identification of potential antigenic candidates (19). Advancements in genome sequencing technologies and biomedical computing sciences have enabled the development of numerous computational and statistical tools and specialized databases to analyze, predict, and annotate various aspects of vaccinology (20). The development of a vaccine against *M. phocimorsus* is particularly warranted over that against other pathogens for several compelling reasons such as the increasedpotential for zoonotic transmission, the inability of humans to develop immunity to *M. phocimorsus* due to limited exposure in the general population and the ability of the pathogen to cause life-threatening bloodstream infections, septicemia, and organ failure (21).

We employed immunoinformatics and simulation approaches using five representative strains to identify potential vaccine targets against *M. phocimorsus*. Following subtractive genomics, we identified a putative immunoglobulin-blocking virulence protein as a potential vaccine candidate with highly antigenic characteristics, and met the criteria of physico-chemical, structural, allergenicity, and antigenicity features, which were further confirmed by docking and molecular dynamics simulation.

## 2 Materials and methods

**Figure 1:**
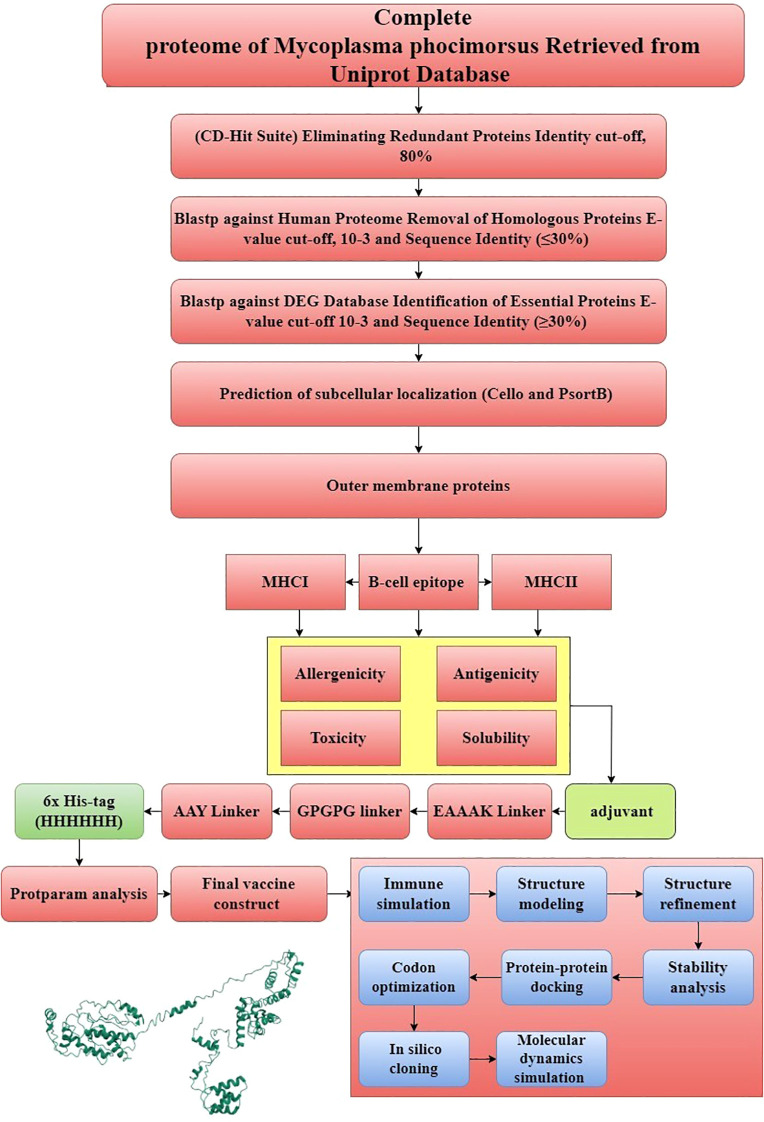
Schematic presentation of the subtractive genomics and reverse vaccinology approaches for potential vaccine development.

**Figure 1** shows a simplified illustration of the chimeric vaccine design and analysis using an immunoinformatic method.

### 2.1 Obtaining proteome data and identifying nonredundant proteins

Six completely sequenced genomes of *M. phocimorsus* (M6620, M6447, M6879, M6642, M5725, and M6921) were retrieved from the NCBI database (**Table 1**) to identify their core proteome (13). The bacterial pangenome analysis (BPGA) tool, a high-speed and robust tool specifically designed for the comprehensive pan-genome analysis of bacterial species, was used for this analysis. BPGA effectively categorizes genes into core, accessory, and unique gene pools, providing critical insights into genetic conservation and variability within and across bacterial genomes. Determining the core proteome is important for choosing proteins or epitopes that are conserved and could be used to develop cross-protective vaccines (22).

**Table 1:** List and features of the genomics data utilized in this study.

Species	Sequencing depth	Contigs	Size	GC content	CDS	Isolation country	Host name
*M. phocimorsus* strain M6447	91.0x	12	744321	25.10906	626	Finland	*Homo sapiens*
*M. phocimorsus* strain M6620	118.0x	51	772409	25.17734	675	Denmark	*Homo sapiens*
*M. phocimorsus* strain M6879	110.0x	12	745732	25.09816	628	Norway	*Homo sapiens*
*M. phocimorsus* strain M6921	140.0x	50	758898	24.96554	662	Sweden	*Homo sapiens*
*M. phocimorsus* strain M6642	126.0x	34	752889	25.11087	641	Denmark	*Homo sapiens*
*M. phocimorsus* strain M5725	94.0x	28	755408	25.17183	641	Denmark	*Homo sapiens*

Moreover, by focusing on the core proteome, we aimed to overcome the antigen variability that is common in *Mycoplasma* species and increase the chances of obtaining broad-spectrum protection. The complete proteomes of the six *M. phocimorsus* strains were obtained from the Universal Protein Resource Knowledgebase (UniProtKB), followed by redundant protein elimination via the CD-HIT suite (23). The criterion for sequence identity was set at 80%, and the remaining parameters were maintained at their default values. A cut off of 80% in CD-HIT influences the selection of nonredundant proteins by clustering sequences that share 80% or more sequence identity into the same group. BLASTp was performed against the human proteome using a list of nonredundant proteins to eliminate proteins that are similar to those identified in humans (24). A bit score cut off of 100, an E-value threshold of 10^-3^, and a minimum sequence identity of ≤30% were set as a criterion.

### 2.2 Prediction of essential proteins and their subcellular localization

Essential proteins were identified using the Database of Essential Genes (DEG) (25), where *M. pneumoniae* used as the reference strain and by using BLASTn algorithm. Essential genes are identified using unique DEG identification numbers, reference numbers, functions, and sequences, and are stored and processed via MySQL as the database management system. The identification process targeted the host non-homologous list of proteins to filter essential proteins. A stringent selection criterion was built, setting a minimum identity threshold of ≥30% and a bit score of 100, to ensure the presence of sequences that are not similar to human proteins. Moreover, nonhomologous sequences were further accurately chosen for subsequent analysis to alleviate potential cross-reactivity. By utilizing PSORTb (26) and CELLO (27) nonhomologous proteins can be further categorized into cytoplasmic and outer membrane (OM) proteins.

### 2.3 Prioritization of antigenic proteins and epitope mapping

The host immune system identifies a molecule known as an antigen, which triggers the immune response (28). The VaxiJen v2.0 server was employed to predict the potential antigenic properties of the selected proteins (29) by setting the cut off to >0.5. We employed AllerTOP v2.1 to assess the allergenicity of the OM protein. To develop an epitope-based vaccine, it is very important that the chosen epitopes can cause a strong and specific immune response (30). OM protein sequences were used to predict the immunogenic epitope via the online IEDB analysis tool (31, 32). VaxiJen v2.0 server, ToxinPred2 (33), and AllergenFP (34) were employed to examine all the potential epitopes along with their respective antigenicity, toxicity, and allergenicity. Only epitopes identified as antigenic, non-toxic, non-allergenic were selected for subsequent analysis.

### 2.4 Vaccine construct design and structure prediction

Linkers significantly contribute to the design of multiepitope vaccines by maintaining structural integrity and enhancing the immunogenicity of epitopes (35). By utilizing different linkers such as GPGPG, highly promising epitopes can be joined. Furthermore, the EAAAK linker was employed to attach the adjuvant, thereby improving the overall efficacy of the vaccine construct. The His-tag, comprising six histidine residues, was subsequently attached to the construct’s C-terminus as previously reported (36). ProtParam (37) performs calculations on the basis of amino acid one-letter codes (A-Z) and ignores non amino acid symbols or average hydropathy scores. PSIPRED web servers (31), which retrieve the protein’s secondary structure providing information on alpha-helices, beta-strands, and coils, were utilized to envisage the physico-chemical attributes, and the secondary structure of the vaccine was subsequently improved by using the GalaxRefine server (38). Both before and after refinement, Z score and Ramachandran plots were used to check the quality of the vaccine model and its structure with PROCHECK and ProSA-web (LaskowskiMacArthur and Thornton; 39).

### 2.5 Molecular docking analysis

Many biological molecules depend on the interaction of proteins for function. To estimate the molecular interactions or affinities between these molecules, analyzing the complex structures that form between them is crucial (40). Toll-like receptors (TLRs), regarded as recognition receptors, largely depend on the recognition of infections. There are ten TLR-encoding genes (41) and among them, TLR-4 was selected to dock with the vaccine construct since it is a useful bacterial-sensing TLR (42). The key interaction between the vaccine model and TLR-4, an innate immune receptor, was examined using the ClusPro 2.0 protein-protein docking server. This particular tool is known for its remarkable efficiency, such as not only providing unusual customization choices, but also rotating the ligand 70,000 times to find the position with the lowest RMSD (43). To ensure that the TLR-4 structure was ready for docking, ligand and oligosaccharide molecules were removed following energy minimization. The ClusPro web server rigid-body docks two proteins through billions of different conformations. The cores of the largest clusters are used as possible models of the complex based on low-energy docked structures. The study revealed the crystal structure of TLR-4, which was obtained from the PDB with the ID 2z64. Protein-protein interactions were examined using the software program RING 3.0 (44).

### 2.6 Molecular dynamics simulations of vaccine constructs

Desmond software facilitated 100-nanosecond molecular dynamics (MD) simulations, enabling a thorough investigation of receptor and ligand complex dynamics by docking studies. This static technique provides a precise image of molecule binding within the protein’s active site (45). We used MD simulations to study the dynamic characteristics of complexes, applying Newton’s classical equation of motion. This approach provides a comprehensive understanding of receptor-ligand systems, allowing for realistic predictions and insights into dynamic interactions in biological settings (46). We extensively utilized Maestro’s protein preparation wizard for optimization and structural refinement. Subsequent systems were meticulously crafted using the System Builder tool. The OPLS_2005 force field and incorporated the TIP3P (transferable intermolecular potential with 3 points orthorhombic box solvent model was employed for simulation. To simulate physiological conditions, 0.15 M sodium chloride was added and counterions were introduced to neutralize the system. The simulations were performed using default parameters to ensure consistency with normal physiological conditions. Specifically, the NPT ensemble was employed, maintaining a pressure of 1 atm and a temperature of 310 K. We relaxed the models before the simulation to establish a stable starting configuration. The simulation trajectories were saved after every 100 ps intervals to ensure a comprehensive analysis. Stability was further assessed by continuously monitoring the root mean square deviation (RMSD) of the ligand and protein over time (16).

### 2.7 Normal mode analysis of the vaccine construct

Protein-protein complex stability is typically assessed via normal mode analysis (NMA), a standard procedure in computational research (47–49). This assessment comprises analyzing the dynamics of proteins and contrasting their behavior with their usual behavior (47) and the iMODS server is a potential tool for this specific purpose (50), as it provides valuable insights into the intrinsic motions of a variety of proteins (51). It computes several parameters, such as B-factors, eigenvalues, covariance, and deformability, to describe the stability and dynamics of the protein (52). Eigenvalues signify the primary chain deformability. This feature is directly related to the energy required to initiate such deformations. Covariance offers information about the coordinated motion of various protein elements by assisting in the identification of protein structure associated movements (53, 54). Elevated B-factors indicate enhanced flexibility and movement. These values are crucial for determining whether the complex performs its intended function and exhibits the expected behavior, particularly in the context of vaccines or other biomedical applications (55, 56).

### 2.8 The vaccine construct, *in silico*, cloning and codon optimization

Once cloned and inserted into an appropriate vector, the *in silico* cloning process ensures that a specific host will express the vaccine construct. To achieve successful cloning, the optimization and vaccine construct insertion into the expression vector were carried out. The Java codon adaptation tool (JCat) was used for the back-translation of vaccine sequences into cDNA to amplify the engineered vaccine. This specific tool plays an essential role in the determination of the codon adaptation index (CAI), the DNA sequence, and the GC content, which are crucial for optimizing the nucleotide sequence (57, 58). Furthermore, SnapGene, a sequence analysis and molecular cloning software built by Insightful Science, was employed. With the help of SnapGene, biologists may easily see, examine, modify, and share data on molecular biology sequences and processes (59).

### 2.9 Immune response simulation

The C-ImmSim platform was used to assess the potential immunological response of the designed vaccine. This process involved the use of a position-specific scoring matrix, with all existing options configured to their default settings. This study simulated the following parameters: a) a vaccine devoid of LPS, b) three vaccine doses were administered at intervals of 1, 84, and 168 days to elicit an effective and enduring immune response, and c) the simulation volume and steps were set to 10 and 1100, respectively. All other parameters remained constant (58).

## 3 Results and discussion

### 3.1 Retrieval of the core proteome and prioritization of vaccine candidates

Six well-known representative and pathogenic strains of *M. phocimorsus* from various countries were selected for vaccine design (**Table 1**), and 3,576 core proteins were retrieved by proteomics analysis. CD-HIT clustering at a 90% threshold yielded 829 nonredundant proteins. Owing to their substantial contribution to the essential functions and pathways of pathogens, unique and non-redundant proteins are promising targets for vaccine design (60, 61).

### 3.2 Non-homologues and essential protein prediction

Homologous proteins share similarities due to their evolutionary origin; therefore, eliminating human homologous proteins from the bacterial proteome is crucial to prevent potential interference (62). A BLASTp analysis identified 192 nonhomologous proteins to the human proteome, reducing the risk of autoimmune responses and improving the safety of the profile for further investigation (63). Essential proteins are vital for the sustainability of cellular functions and constitute a core set of proteins that are crucial for life support (63). BLASTp analysis revealed 170 proteins as essential proteins. These essential proteins appear to have the ability to modulate important processes such as pathogenicity, nutrient absorption, and virulence.

### 3.3 Assessment of subcellular localization and physico-chemical properties

We retained the essential proteins identified as OM for further analysis, a crucial step in assessing their suitability for vaccine design. Two proteins, the C1 family peptidase and the putative immunoglobulin-blocking virulence protein, were identified as OM via the online tools CELLO v. 2.5 and PSORTb v.3 (**Table 2**) and were considered suitable for subsequent assessment as potential vaccine candidates. OM proteins are chosen because of their exposure to the host’s immune system and their antigenic sequences, which trigger strong immune responses (60). Among these two proteins, the putative immunoglobulin-blocking virulence protein with a molecular weight of 60701.75 Da, which contains 540 amino acids, has an isoelectric point (pI) of 9.15 because its pH is greater than neutral at 7.0. This protein resembles positively charged proteins and has an instability index of 37.15 indicating nucleoprotein stability. Additionally, the protein has high thermostability as evidenced by its aliphatic index of 79.26. The ability of the putative immunoglobulin-blocking virulence protein to meaningfully contribute to infection and replication within host cells is well established, and this protein is also used in vaccine design (64).

**Table 2:** Outer membrane proteins identified as potential vaccine candidates.

Accession no.	Protein name	Sequence	VaxiJen score	Antigenicity
UPI0024BF7784	C1 family peptidase	MKKRIAAIYAFLGLVSLSGASMHYHSNKYVFHESVYDPRTMTLNNYLTKVKHQGKDGICWAYSTTAVIESNILKNKLAIDPLNLDLSEKNLAYKTLNRLTNEDVLHNSDFDNYTNNNWLNEGSRTIFAGIASLQWNKLKRESENWQTALDLNDYKVTDYISLNHLKENWKQNVKTAIKAYGAVSISFDISNIYKKLYYNPNELSANVKFPHAATIVGWNDNIEANKFGHKTKTNGAWIVKNSWGDKFGEGGYFYLSYEALIQDLFTLNVVKGDEYTSNYYYDGGYKDMYENQETAHQKATVSFWAKNSLPTLKEKLKAVNVGIFGDDNEVEIKIYKNNNNVTPNSLELGQLVHTQRQHFIHGGLRTIILDNPIYLEPNENFSIVAQILNPKGYSAIRFSKESNSQSDFSYIEENGKWVSSQKHLDGAVARIKAFVVTEKVQNQEESNDLKYAKVILKGKYYHQYNEKVDEELITVMYKDKKLELNKDYTLQYTTEIDENIFKHSKASVGYTKVKINGTGTYTGNNFIFLDVKKANKNSYK IKLEENWKMLSNKGIYYGLNRIEIEYIGPNKHLFNNNKVTLNIHKIDPNKPKEEPIIIKNEDKTWNSLFSALSKFGQIIVSFFSFW	0.4216	ANTIGEN
UPI0024BFD15D	putative immunoglobulin-blocking virulence protein	MKRKNRIILFITSFSVLPIATTTGYFIYKHFLNDNKTHIFESNDNKLQNNARQQNSNKIYIADINFKENIPKLPPRKEPLPIEIPNSNKTINILENKLTPFIPKKEKEKLQRITKVPDLIIKPSEIPTPKPNPKPASPTPAPIPTPPTPKPAPIPVPAPSIPSPAPTPEPSPVPSPAPSNENISGGLYEESADSTGGNYGTGNYTGWDKSGNQLEEGKQIKGSEFFKYGVSTKNHEIKIFKLEDKNNKNILGIKNGYSVDADLSNVFGLTTFRSVNELLDNDNKKVIQYRFTNIGAYDDTRNLKLIFESIQENAPQVTLIFKENRIDMLKHLKNKKIKQLDLFSNSDVNSKNWSINPLFLENISNINNNNYANEIGLDTDTNGGKKIVFNSLYFNKEDVDNDNNKFSKINKGLKMVYEDRKNEDFFKGSKKVGYPTELDLSDTDLKSLKGLKFDFTDSRGKKVRLTKLTLNGGNSSNFEINADELNEANFEVLDYDYSGSEIVFKGNVNKISPKNPNDLTDQGKKNLGILRKLAKISA	0.6144	ANTIGEN

Additionally, immunoglobulins are thought to affect bacterial infections in a number of ways, including antibody-dependent cell-mediated cytotoxicity, complement activation, phagocytosis, and toxin neutralization (65, 66). The processes by which immunoglobulins influence bacterial infections are believed to include bacterial cell destruction by complement activation, phagocytosis via bacterial opsonization, toxin neutralization, and antibody-dependent cell-mediated cytotoxicity (64).The literature reveals that the selection of proteins on the basis of various stringent criteria has potential and is vital for the development of multiepitope based vaccines.

### 3.4 Epitope prediction and analysis lead to the finalization of potential vaccine candidates

Subsequent analysis of the epitopes was conducted through the IEDB platform. Through the utilization of the B cell epitope prediction tool provided by the IEDB, nine peptides were identified as possible B cell epitopes (**Table 3**).

**Table 3:** The table presents the prediction of linear B cell epitopes along with their potential vaccine features.

Start	End	Peptide	Length	Non-allergenic
34	141	DNKTHIFESNDLNKLQNNARQQNSNKIYIADINFKENIPKLPPRKEPLPIEIPNSNKTINILENKLTPFIPKKEKEKLQRITKVPDLIIKPSEIPTPKPNPKPASPTP	108	Yes
147	257	PPTPKPAPIPVPAPSIPSPAPTPEPSPVPSPAPSNENISGGLYEESADSTGGNYGTGNYTGWDKSGNQLEEGKQIKGSEFFKYGVSTKNHEIKIFKLEDKNNKNILGIKNG	111	Yes
267	271	VFGLT	5	Yes
333	334	HL	2	Yes
336	340	NKKIK	5	Yes
346	355	SNSDVNSKNW	10	Yes
429	464	KGSKKVGYPTELDLSDTDLKSLKGLKFDFTDSRGKK	36	Yes
467	501	LNEANFEVLDYDYSG	15	Yes
511	524	NKISPKNPNDLTDQ	14	Yes

**Figure 2:**
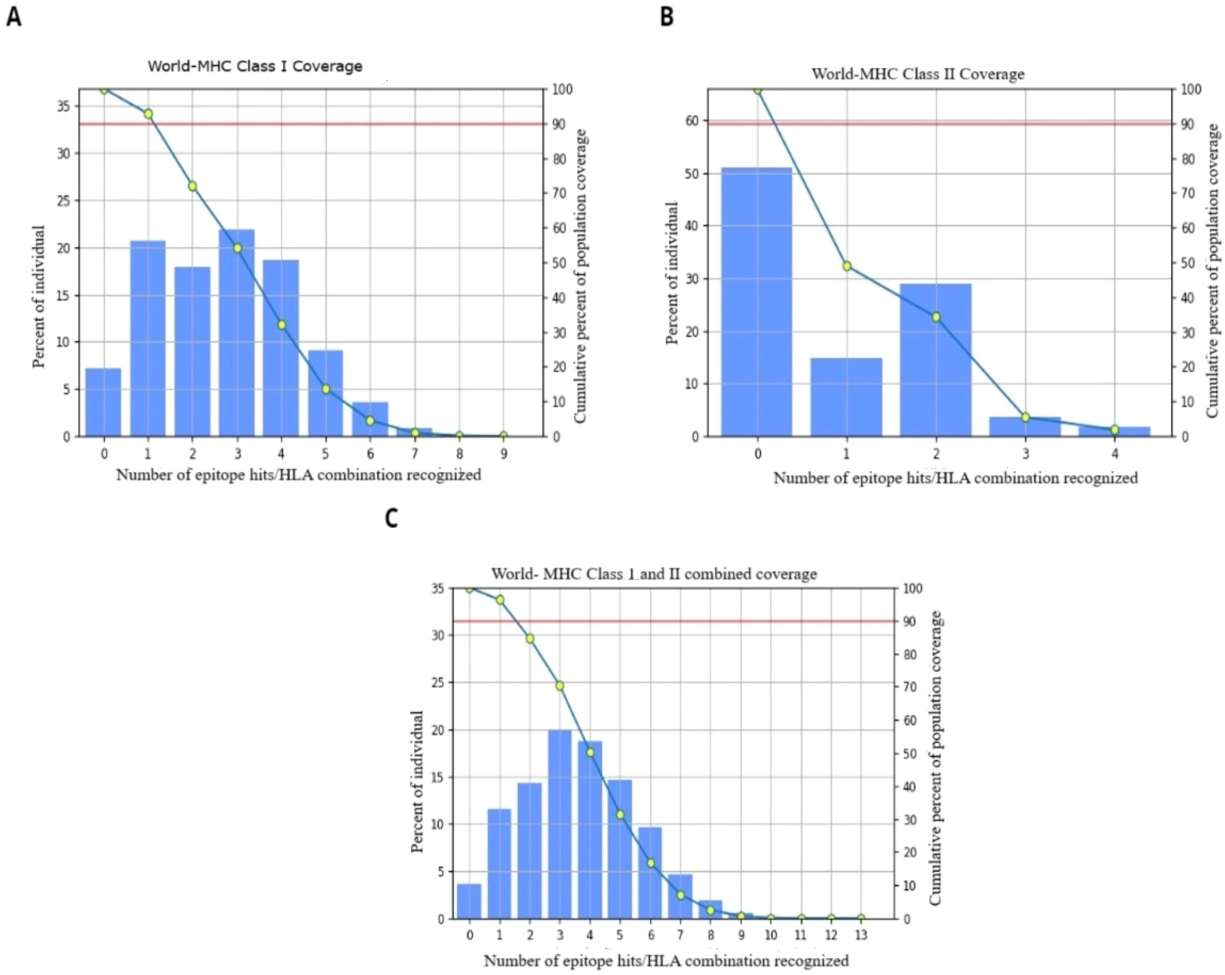
**(A)** Global population coverage of MH class I epitopes. **(B)** Global population coverage of MH class II epitopes. **(C)** HLA allele coverage worldwide for T-cell epitope prediction.

The elimination of bacteria from the body generally requires the involvement of both humoral and cellular immunity mechanisms. A very important part of initiating humoral immune responses is the interaction between B cell epitopes and antibodies (67). The predicted B cell epitopes were analyzed to evaluate their potential immunogenicity and determine their binding affinities and interaction sites with major histocompatibility complex (MHC) class I and class II molecules as shown in **Figure 2**, with a cut off value of 0.5.

B cell epitopes were selected based on several criteria, including their sequence, position, antigenic scores, length, and nonallergenic properties as detailed in **Table 3**. The antigenic scores ranged from a minimum value of 0.4439 to a maximum of 0.6107. Analysis of antigenicity revealed both the highest and lowest values, with a mean score of 0.7926. It is highly useful for identifying specific regions of a protein that are recognized by antibodies. These regions, known as B cell epitopes, are specific segments of an antigen that can elicit an antibody response, enabling the design of targeted antibodies without requiring the entire protein. Following B cell epitope prediction, 170 potential MHC class I epitopes were predicted on the basis of the stringent selection criterion of an IC value less than 1000 via the IEDB MHC class 1 tool. Eight epitopes were selected for inclusion in the vaccine formulation after 63, which fulfilled the pre-requisite of interacting with at least 10 alleles that were eliminated. As shown in **Table 3**, these eight epitopes were chosen because of their potential antigenic properties as well as the lack of any poisonous or allergic characteristics.

The utilization of the IEDB Tongaonkar and Kolaskar antigenicity methods resulted in seven MHC class II epitopes which were categorized by their nonallergenic and nontoxic natures (68). Together with their innate antigenicity, the noteworthy Comb scores of the predicted epitopes make them extremely promising choices. In particular, epitopes such as LKSLKGLKFDFTDSR, GLKFDFTDSRGKKVR, DLKSLKGLKFDFTDS, NLKLIFESIQENAPQ, RGKKVRLTKLTLNGG, LKGLKFDFTDSRGKK, RIDMLKHLKNKKIKQ have been recognized as potential binders with alleles such as HLA-DRB4*01:01, HLA-DRB3*01:01, HLA-DRB3*01:01, HLA-DRB1*07:01, HLA-DRB4*01:01, HLA-DRB1*03:01, and HLA-DRB5*01:01 (**Table 4**).These findings suggest a broad population coverage and highlight the potential of these epitopes to activate CD4^+^ T-helper cells, which play a pivotal role in coordinating both humoral and cellular immune responses (69). Overall, the identification of these highly antigenic, conserved, and nonallergenic MHC class II epitopes underscores their promise as key components for the design of an effective multiepitope vaccine against *M. phocimorsus*.

**Table 4:** Predicted MHC class I with their antigenic score and allergenic and toxic properties.

Epitopes	Interacting alleles	Antigenicity	Allergenicity	Toxicity
APTPEPSPV	HLA-B*51:01	0.8684	NO	NO
DNKKVIQYR	HLA-A*68:01	0.481	NO	NO
EIVFKGNVNK	HLA-A*30:01	0.4079	NO	NO
ELNEANFEV	HLA-A*02:06	1.4901	NO	NO
ESIQENAPQV	HLA-A*26:01	0.8181	NO	NO
FLNDNKTHI	HLA-B*53:01	0.9904	NO	NO
GKQIKGSEFF	HLA-A*23:01	0.9031	NO	NO
HLKNKKIKQL	HLA-A*02:03	1.0732	NO	NO

Furthermore, the integration of multiple peptide epitopes that can activate various HLA-restricted T cell specificities, in conjunction with B cell epitopes, establishes the basis of a universal vaccine formulation. This design guarantees a comprehensive immune response by activating both humoral and cellular immunity. T cell epitopes activate CD4+ and CD8+ T cells across diverse HLA haplotypes, fostering strong and enduring immunity, whereas B cell epitopes augment antibody synthesis, facilitating quick pathogen neutralization (70, 71). This integrated method is especially advantageous for addressing genetic heterogeneity within the human population, hence enhancing vaccine efficacy across various populations.

### 3.5 Population coverage analysis

By employing the IEDB tool, a comprehensive evaluation of global population coverage was conducted for four epitopes, considering MHC class epitopes I and II alleles worldwide. This detailed analysis aims to ensure broad population coverage, supporting the design of a vaccine capable of targeting diverse populations across the globe (72). The study revealed that 92.77% of the global population was covered by MHC class I epitopes and that 49.02% was covered by MHC class II epitopes. These findings indicate that these epitopes could be used to make vaccines, as shown in **Figure 2**. The shared population coverage was 96.31%. Typically, population coverage above 80% is observed for MHC class I epitopes as the class I alleles (HLA-A, HLA-B, HLA-C) are more conserved and fewer in number, whereas MHC class II epitopes (HLA-DR, -DQ, -DP) are more polymorphic, resulting in lower coverage, usually around 50-60% (73, 74). In terms of relevance to vaccine design and epitope prediction, population coverage reproduces the capacity or likelihood of epitopes binding to MHC molecules across diverse global populations (75). Several factors play roles in the target population for a vaccine, such as the disease’s epidemiology, affected demographic groups, and public health priorities (76). Overall, the highly significant percentage of the global population justifies vaccine design.

### 3.6 The proposed MEV model

**Figure 3:**
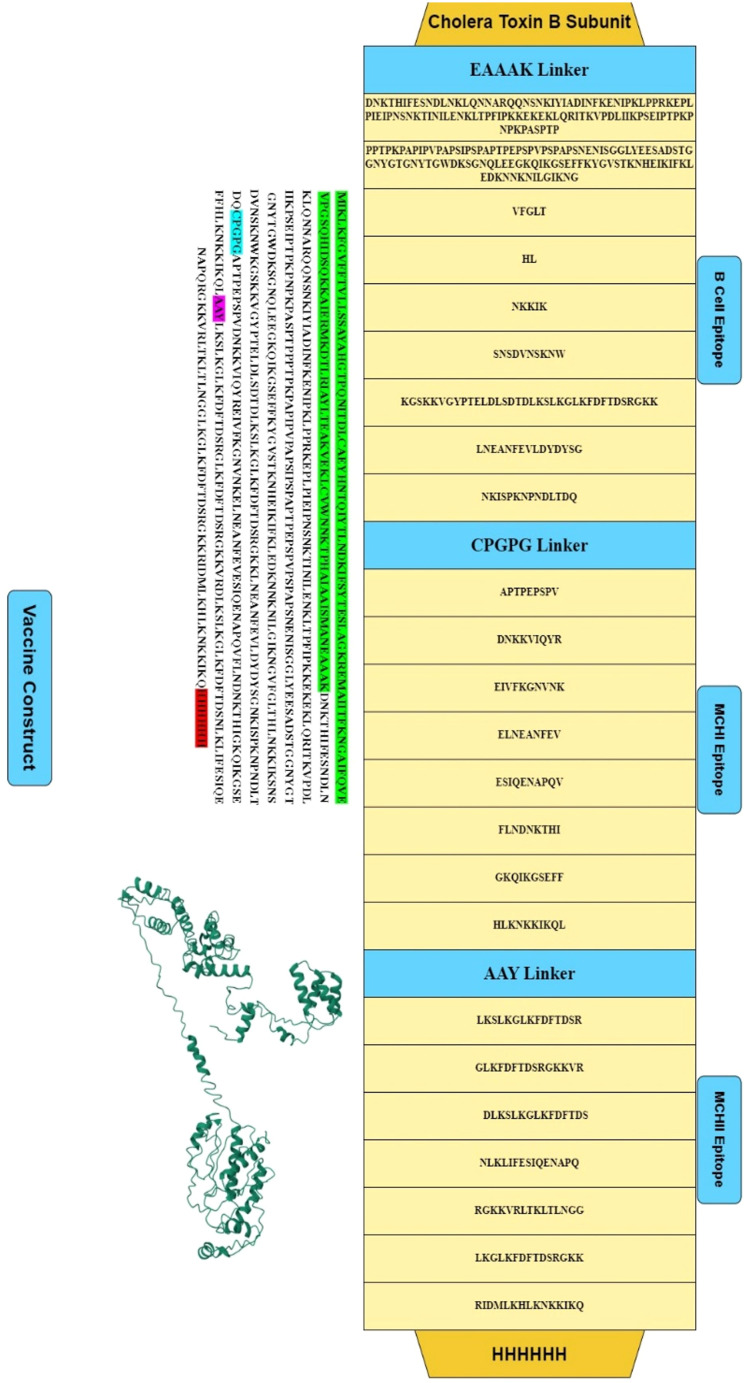
A visual representation of the multi-epitope vaccine construct is depicted through graphics. The components of the construct are linked together using different linkers, namely (1) EAAAK, (2) CPGPG, and (3) AAY. CD8+ epitopes are joined using the AAY linker, CD4+ epitopes are joined using the CPGPG linker, and B-cell epitopes are joined using the EAAAK linker.

Small peptide linkers were used to link 24 shortlisted epitopes i.e., nine B cell epitopes, eight MHC class I epitopes, and seven MHC class II epitopes. **Figure 3** represents the 630 amino acid-based vaccine model in brief MEV (multiepitope vaccine), which was enhanced with an immunogenic adjuvant and extra supporting peptides. An adjuvant used in vaccine design is a protein cholera toxin B subunit, which is used as an adjuvant in universal vaccine formulations and can enhance the immune response against both B cell and T cell epitopes, ensuring broad and long-lasting immunity (77). The cholera toxin B subunit is a powerful mucosal adjuvant that generates mucosal antibody responses and specific immunity (78).

It binds strongly to the GM1-ganglisoside receptor, enhancing the immunogenicity of exogenous antigens, enhancing B cell and T cell responses, and decreasing the antigen dose (79). Various linkers were used to combine adjuvants with B cell epitopes in order to trigger an adapted immune response (80). Five components (the adjuvant, EAAAK linker, GPGPG linker, AAY linker, and 6x His tag) were combined to strengthen the structure, provoke a significant immunological reaction, and facilitate its use in further purification tests. The first step was the introduction of a TLR-4 adjuvant with UniProt ID: 2z64. Subsequently, linkers were utilized to separate and combine the B and T cell-specific epitopes within the construct. We added a 6x His-tag to the vaccine sequence (at the C-terminus) to identify and purify the proteins.

### 3.7 Prediction of the secondary and tertiary structures of the vaccine

The secondary and tertiary structures of proteins are crucial in vaccine design, particularly in protein-based or peptide-based vaccines. The secondary and tertiary structures of the formulated vaccine model included 49.34% helices, 12.57% sheets, and 37.22% loops. The Ramachandran plot specified that 84.2% of the residues of the tertiary structure exist in the most favored region of the refined structure (**Supplementary Figure S1**). We added a 6x His-tag to the vaccine sequence (at the C-terminus) to identify and purify the proteins. The solubility of our MEV vaccine was assessed via protein-sol, a crucial stage in vaccine biotechnology, as it aids in purification and isolation processes. The vaccine protein construct was soluble, when it was overexpressed in *E. coli* (predicted scaled solubility: 0.581), thereby ensuring downstream isolation and purification. Both the tertiary and secondary structures of the vaccine model were characterized by PSIPRED web servers, GalaxRefine server, PROCHECK and ProSA-web and its solubility were in line with those reported previously (41, 58).

### 3.8 Molecular docking studies of the vaccine construct and the TLR-4 receptor

**Figure 4:**
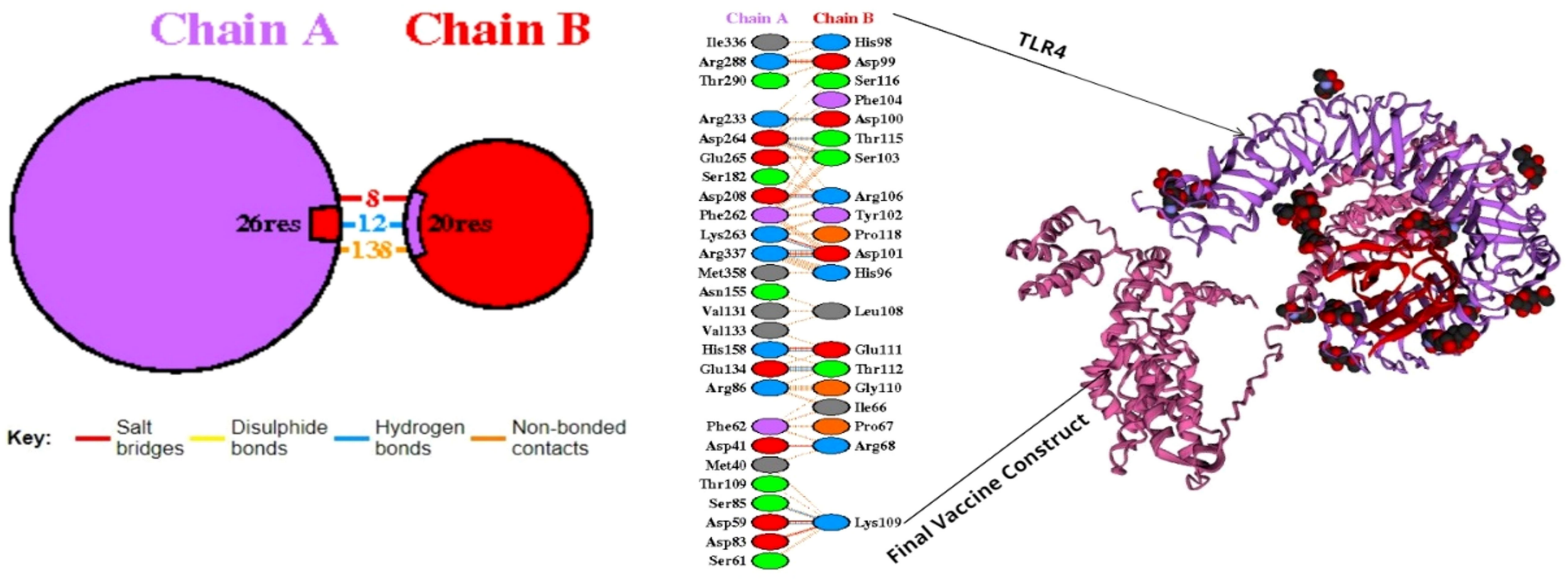
The molecular docking structure of MEV and TLR-4. As illustrated in the figure, “pink” stands for the MEV, and “light purple” stands for the TLR-4 and red color denotes interacting residues.

Molecular docking that accurately enables the interaction between a ligand and a target protein such as TLR-4 is enabled by the use of ClusPro. In total, ten models were obtained, among which the first model encoded a virtuous H-bond interaction and a 90.3 favored region as shown in **Figure 4**. The lowest energy weighted scores are -1383.2 and -1033.0. The H-bond analysis findings revealed that specific residue pairs interacted with each other, including ASP59-LYS109 with a distance of 2.85 Å, SER85-LYS109 with a distance of 3.21 Å, GLU134-THR112 with a distance of 3.09 Å, and GLU134-THR112 with a distance 3.19 Å. Additionally, HIS158-GLU111 interacted with a distance of 3.01 Å, ASP208-ARG106 with a distance of 3.29 Å, ARG233-ASP100 with a distance of 2.65 Å, LYS263-ASP101 with a distance of 2.82 Å, ASP264-THR115 with a distance of 3.27 Å, ASP264-SER103 with a distance of 2.64 Å, ARG337-ASP101 with a distance of 2.70 Å, and ARG337-ASP101 with a distance of 2.80 Å. The use of PDBsum to represent different interactions between the TLR-4 receptor and the vaccine construct can indeed provide valuable insights into the immunogenic potency of the vaccine.

### 3.9 Vaccine-TLR-4 complex MD simulations

**Figure 5:**
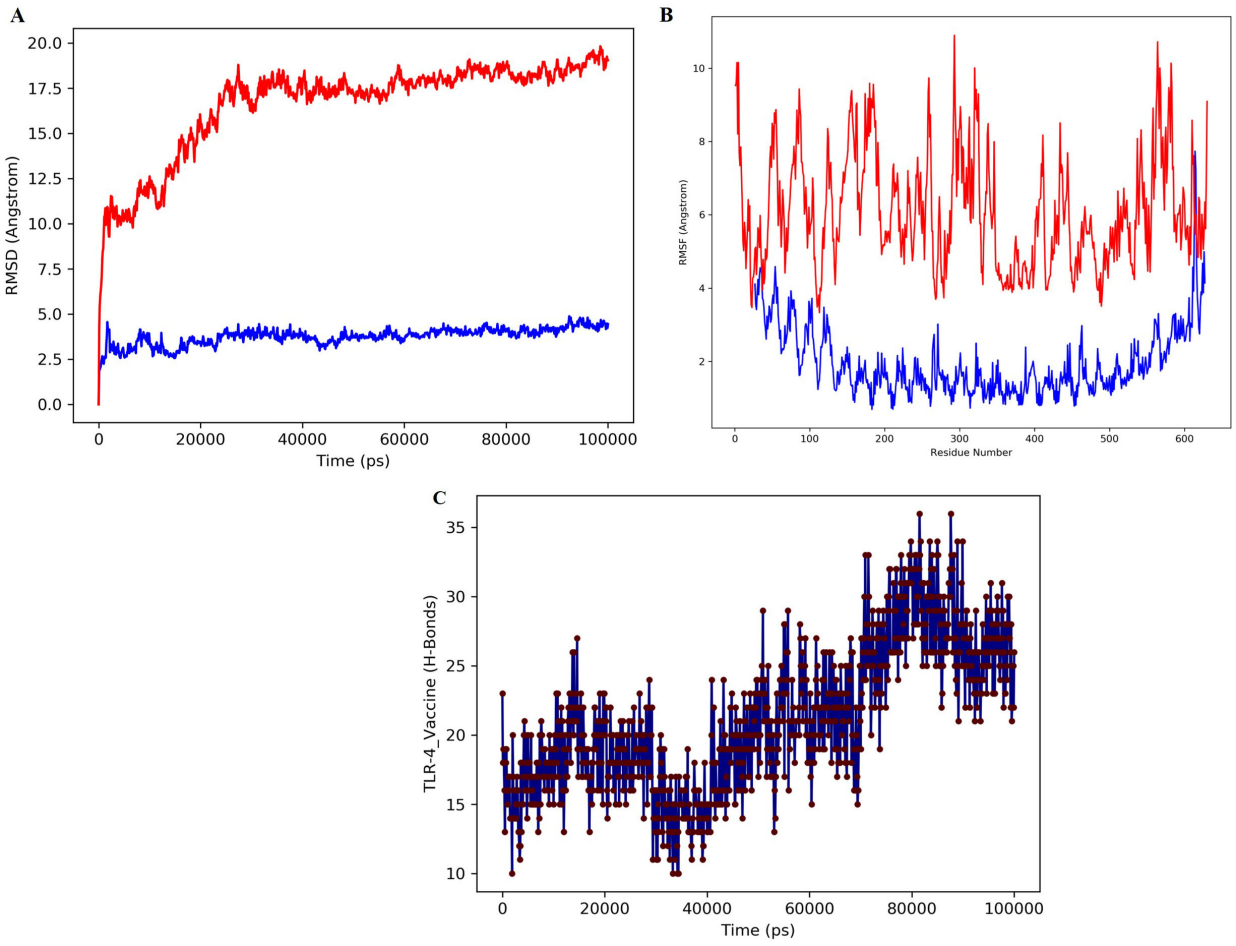
MD simulation of the docked complex of MEV, **(A)** The structural stability of the protein over time of the vaccine construct. The blue color represent receptor protein RMSD while red color denotes the design vaccine RMSD. **(B)** RMSF assesses the flexibility of individual residues of the vaccine construct. The blue color represents the receptor protein RMSF while red color represents the vaccine RMSF. **(C)** Represent the number of hydrogen bonds formed between the TLR-4 receptor and the vaccine construct.

The vaccine-TLR-4 complex was dynamically simulated for 100 ns in the MD simulation to confirm the stability of the complex in a dynamic state. Various analyses, such as RMSD, RMSF, and H bond analyses were carried out for the MD simulations. The analysis of macromolecular structures and dynamics widely utilizes RMSD. Two basic parameters can be understood based on the pattern of the RMSD plot: a) whether the system had reached the equilibrium state, and b) whether the simulation time was sufficient. The RMSD plot of the simulated complex reached a plateau, confirming system equilibration and indicating that the simulation time was sufficient for this protein under the given condition (**Figure 5A**). Furthermore, the absence of significant fluctuations in the RMSD profile demonstrates the stability of the ligand–receptor complex throughout the simulation.

The fluctuation of each protein residue was analyzed by using RMSF (**Figure 5B**). Figure B shows the RMSF profile of protein residues during the simulation. The vaccine–TLR-4 complex (blue) displays consistently lower fluctuations approximately 1-3 Å than the unbound structure (red), which reaches 8-10 Å in several flexible regions. This reduction in residue mobility indicates that vaccine binding stabilizes the receptor, particularly in loop and terminal regions. Overall, the RMSF trend confirms that the complex maintains a more rigid and stable conformation throughout the simulation. Intermolecular hydrogen bonds of the vaccine and TLR-4 were observed throughout the time of simulation. **Figure 5C** represents a significant number of hydrogen bonds. These results indicate that the receptor-ligand complex is compact and that the components are moving closer and forming more stable interactions. Overall, the final construct-TLR-4 complex demonstrated good stability in a solvated dynamic state.

### 3.10 Normal mode analysis of potential vaccine candidates

**Figure 6:**
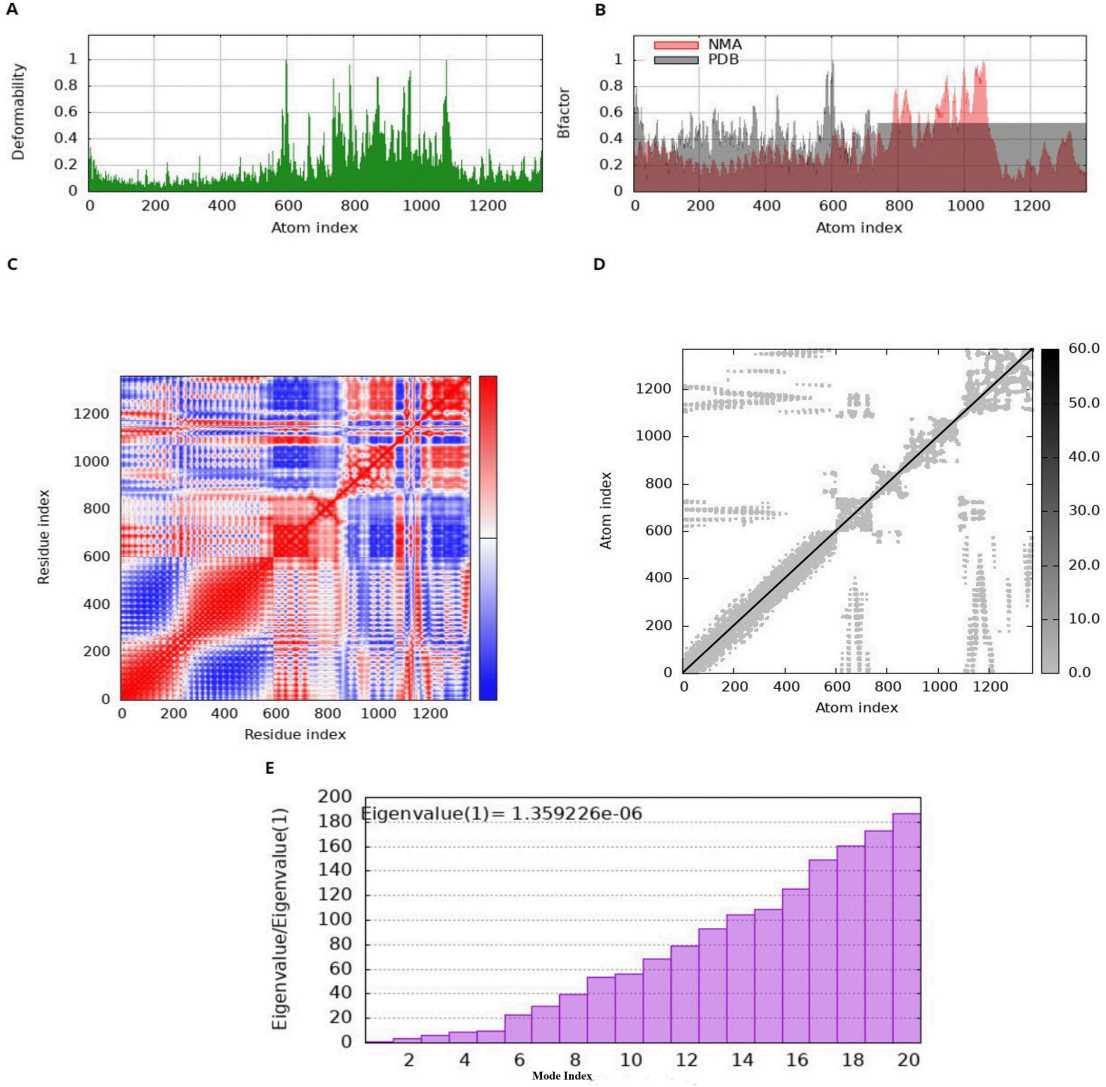
Normal mode analysis of MEV, **(A)** deformability representing defined feature of *M. phocimorsus* that plays a critical role in its pathogenicity and immune evasion **(B)** B-factor; reflecting the atomic mobility or flexibility within a biomolecule **(C)** covariance index representing the conserved regions and binding interfaces that are less prone to mutation, making them ideal targets for vaccines, **(D)** elastic network analysis representing the potential protein dynamics and interactions useful for vaccine design. **(E)** A bar chart of eigenvalue ratios against mode index, with a noted eigenvalue.

The normal mode behavior of the MEV and receptor docked complex was assessed using iMODS, which provides important information about the deformability of particular residues. Deformability, in this context, pertains to the propensity of a protein to undergo conformational changes in its three-dimensional configuration. The graphical illustration of peaks represents areas of high deformability, which shows how easily the protein’s three-dimensional structure can be changed (81). Specifically, the NMA data exhibit higher and more frequent peaks compared to the PDB data., indicating that NMA forecasts higher B-factors. This illustrates, on the other hand, that experimentally calculated B-factor values from the PDB file show less flexibility and mobility than those anticipated by computational NMA simulations. Moreover, the variance graph shows an inverse relationship with the eigenvalue graph, with red indicating individual variance and green representing cumulative variance. The covariance matrix shows the relationships between residues with white, blue, and red colors representing uncorrelated, correlated, and anticorrelated interactions among amino acids respectively (81). The stability of the docked complex is shown by examination of the docking complex, which reveals a strong correlation between residue pairs (**Figure 6**).

### 3.11 C-ImmSim simulation

**Figure 7:**
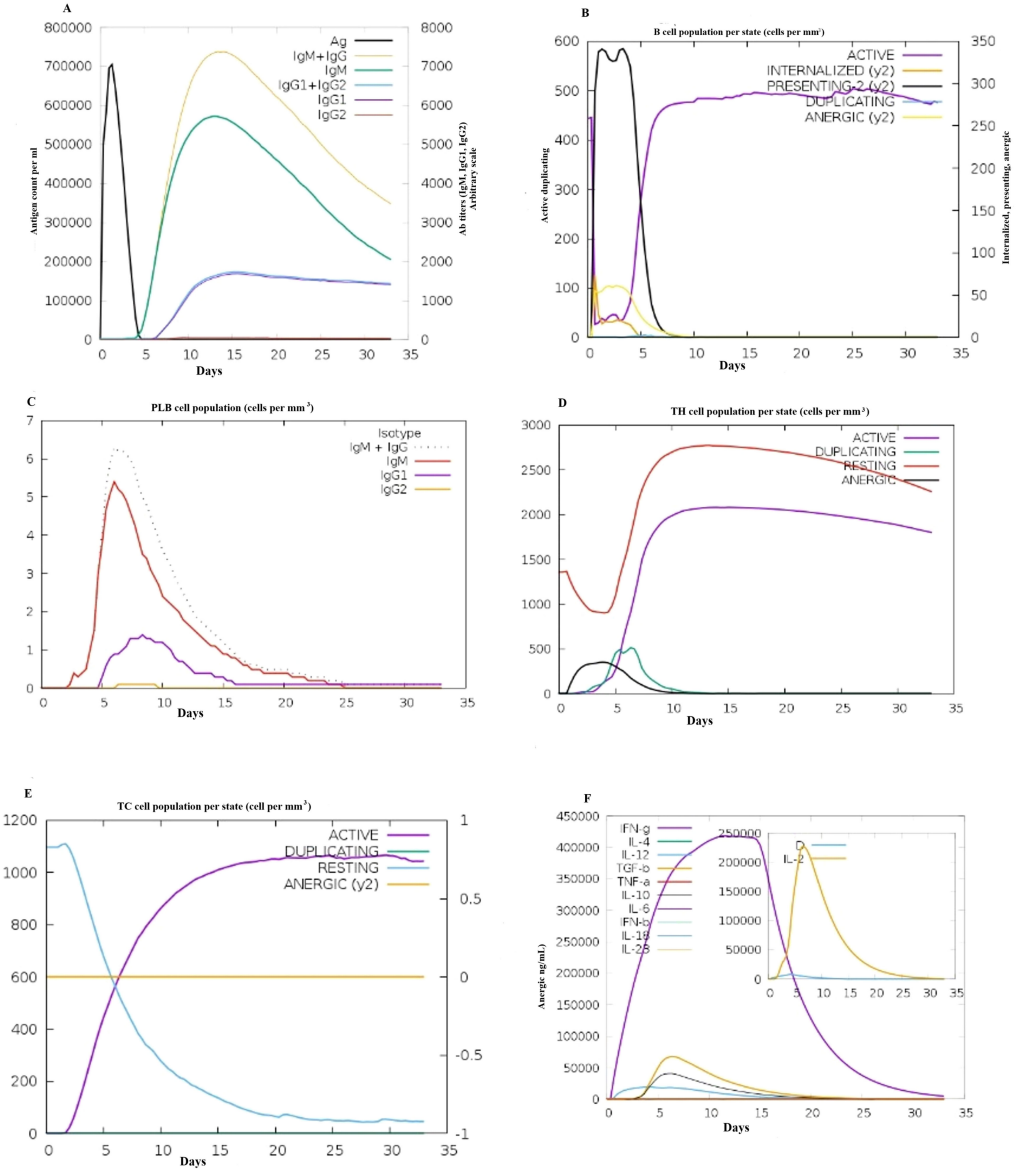
Immune simulation profile of the designed vaccine construct **(A)** Immunoglobulin production shown by black lines after antigen injection; colored lines indicate immune cell classes **(B)** Changes in B-cell population and memory production **(C)** Total number of B-lymphocyte cells in plasma per cell **(D)** Production of helper T-cells **(E)** Total number of TC cells **(F)** Displayed elevated rates of cytokines and interleukins.

Importantly, according to the C-ImmSim immunological tool, the antigen count of the injected antigen peaked count on the fifth day after injection, followed by a slow decline until the fifteenth day. Subsequent to antigen introduction, several antibodies increased (IgM > 600,000; IgG + IgM > 700,000; IgG1 > 500,000; IgG1 + IgG2), and the concentration of the antigen decreased. The data in **Figure 7A** show a substantial increase in the ratios of the IgM and IgG titers. Additionally, as shown in **Figure 7B**, there was an obvious increase in B-cell counts following each vaccination session. Interestingly, T cell activity significantly increased after primary and secondary immunization, and this increase was amplified in later stages, as shown in **Figures 7C, D**. Notably, the impact of the vaccine formulation on innate immune cell populations is shown in **Figure 7E**. Moreover., the significant increase of T helper cells (TH) cells and the levels of IFN-γ, and IL-2 play pivotal roles in the immune response, as shown in **Figure 7F**.

### 3.12 Codon optimization and *in silico* cloning

**Figure 8:**
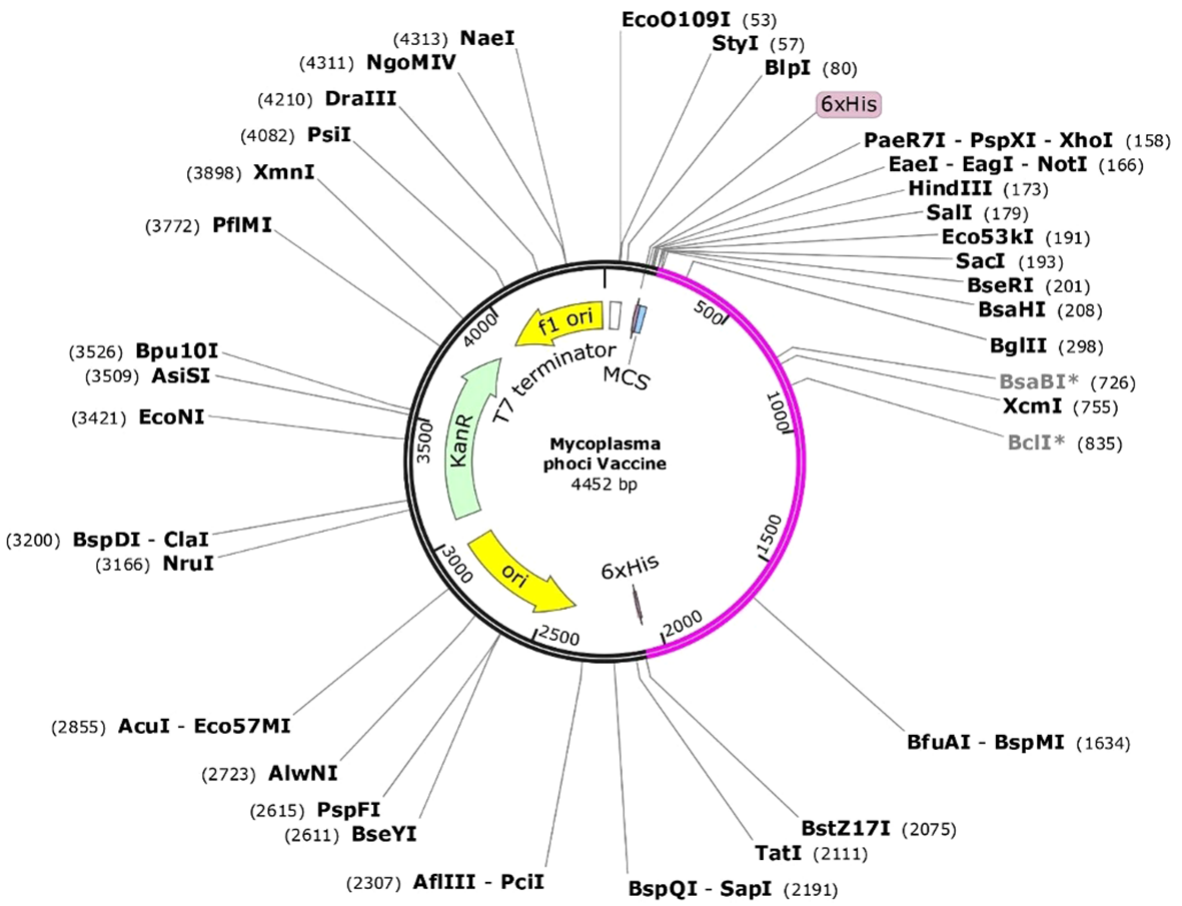
The codons optimized for the gene linked to the vaccine protein were computationally inserted into the pET28a (+) vector within microbial platforms to enhance expression. Integration of the genetic sequence into the cloning vector occurs at the multiple cloning site. The genetic sequence of the tailored vaccine construct (depicted in Magenta), the foundational structure of the vector (depicted in Black), and the kanamycin resistance gene (depicted in Green). Direction and location of gene expression are indicated by colored arrows.

The codon optimization process was carried out via the JCat program (82), and the final vaccine was cloned. To obtain the highest level of expression in strain K12 of E. coli, the sequence of the vaccine was reverse translated. An average CAI value of 54.8% and a predicted GC content of 0.95 were found when the final vaccine model was assessed. These results show that the vaccine design was successfully expressed in the E. coli system, indicating that the expression procedure was carried out successfully (**Supplementary Figure S2**). The optimized codon sequence of the finished vaccine was then included in the pET28a (+) vector using the SnapGene tool to create the recombinant plasmid (**Figure 8**). Typically, codons are commonly assessed using the CAI, a scale ranging from 0 - 1. A CAI value of 0 indicates that synonymous codons are used equally within a gene, whereas a value of 1 reflects a strong preference, utilizing only the best one.

## 4 Conclusions

The comprehensive analysis and design efforts focused on various representative *M. phocimorsus* strains have achieved promising results in the pursuit of treatment and preventive measures against this bacterium. Through in-depth genome and proteome analysis, a potential vaccine candidate has been identified, emphasizing the importance of understanding the biology and pathogenic mechanisms of bacteria. In summary, the integrated approach outlined here represents a potential vaccine construct to fight against *M. phocimorsus* infection, offering promise for effective treatments involving protein-based vaccines and mitigating the impact of this emerging pathogen on human health. Moreover, since the vaccine was designed using core and conserved proteins from six strains of *M. phocimorsus*, it may offer broader applicability by protecting against infections caused by related *Mycoplasma* species, particularly those sharing common antigens or conserved virulence factors. Additional experimental validation and clinical studies are essential to confirm the efficiency and safety of the designed vaccine, ultimately paving the way for its clinical application.

## Data Availability

The original contributions presented in the study are included in the article/**Supplementary Material**. Further inquiries can be directed to the corresponding author.
