# Supplementary figures and images for: An integrated immunoinformatic approach to design a novel multiepitope chimeric vaccine against Mycoplasma phocimorsus as a causal agent of bloodstream infections
Source: Front Immunol. 2025 Dec 5;16:1719398. doi: 10.3389/fimmu.2025.1719398 (PMC12714920; doi:10.3389/fimmu.2025.1719398)

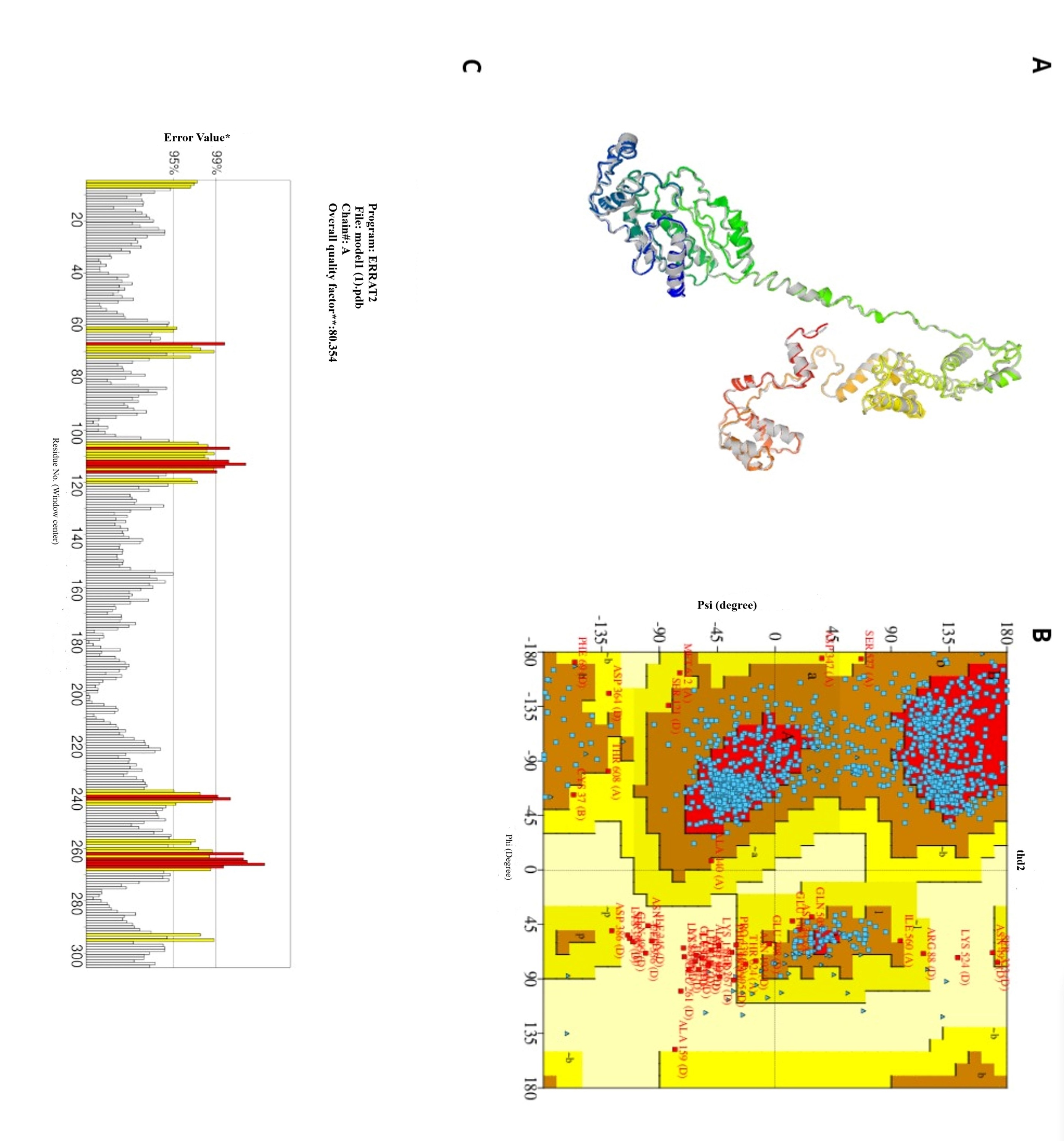

Supplement: Supplementary file 1 [file Image1.jpeg]

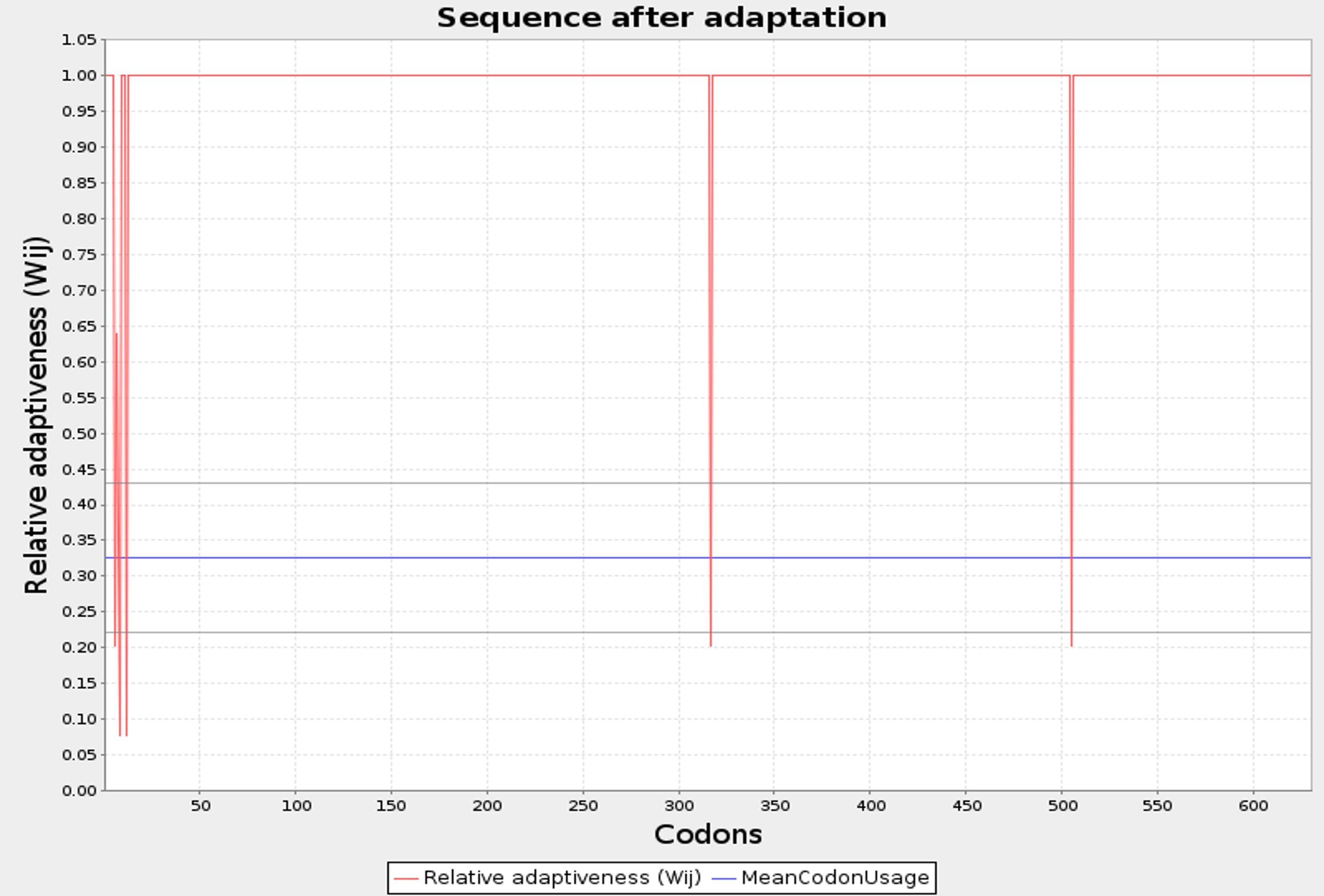

Supplement: Supplementary file 2 [file Image2.jpeg]
